# Lipopolysaccharide-induced changes in the neurovascular unit in the preterm fetal sheep brain

**DOI:** 10.1186/s12974-020-01852-y

**Published:** 2020-05-28

**Authors:** Clémence Disdier, Fares Awa, Xiaodi Chen, Simerdeep K. Dhillon, Robert Galinsky, Joanne O. Davidson, Christopher A. Lear, Laura Bennet, Alistair J. Gunn, Barbara S. Stonestreet

**Affiliations:** 1 Department of Pediatrics, Women & Infants Hospital of Rhode Island, Alpert Medical School of Brown University, 101 Dudley Street, Providence, RI 02905 USA; 2 Department of Physiology, The University of Auckland, Auckland, New Zealand

**Keywords:** Cortex, Fetal brain, Inflammation, Lipopolysaccharide, Neurovascular unit, Sheep, White matter

## Abstract

**Background:**

Exposure to inflammation during pregnancy can predispose to brain injury in premature infants. In the present study, we investigated the effects of prolonged exposure to inflammation on the cerebrovasculature of preterm fetal sheep.

**Methods:**

Chronically instrumented fetal sheep at 103–104 days of gestation (full term is ~ 147 days) received continuous low-dose lipopolysaccharide (LPS) infusions (100 ng/kg over 24 h, followed by 250 ng/kg/24 h for 96 h plus boluses of 1 μg LPS at 48, 72, and 96 h) or the same volume of normal saline (0.9%, w/v). Ten days after the start of LPS exposure at 113–114 days of gestation, the sheep were killed, and the fetal brain perfused with formalin in situ. Vessel density, pericyte and astrocyte coverage of the blood vessels, and astrogliosis in the cerebral cortex and white matter were determined using immunohistochemistry.

**Results:**

LPS exposure reduced (*P* < 0.05) microvascular vessel density and pericyte vascular coverage in the cerebral cortex and white matter of preterm fetal sheep, and increased the activation of perivascular astrocytes, but decreased astrocytic vessel coverage in the white matter.

**Conclusions:**

Prolonged exposure to LPS in preterm fetal sheep resulted in decreased vessel density and neurovascular remodeling, suggesting that chronic inflammation adversely affects the neurovascular unit and, therefore, could contribute to long-term impairment of brain development.

## Background

Exposure to inflammation and/or infectious agents resulting from premature rupture of membranes and chorioamnionitis during gestation is highly associated with adverse neonatal sequela including chronic lung disease, adverse neurodevelopmental outcomes along with long-term disabilities [1–3]. Premature infants surviving after exposure to inflammation around the time of birth are at high risk for a wide spectrum of adverse neurodevelopmental outcomes, presumably, because of injury to the developing brain [1–7]. Moreover, they are at continued risk for disability throughout childhood [6–9]. Lipopolysaccharide (LPS) is a gram-negative bacterial endotoxin that is widely used as a robust inflammatory catalyst to stimulate neuroinflammatory responses in a variety of animal models including fetal sheep [10–15].

A complex network of blood vessels is required to provide nutrients and oxygen to the developing brain. The neurovascular unit (NVU) integrates cell interactions between brain endothelial cells, the basal lamina, and the surrounding glial limitans and pericytes [16]. Each component of the NVU is intimately interconnected with the other constituents to establish an effective anatomical and functional system [17]. This unique complex system maintains brain homeostasis, preserves the integrity of the blood-brain barrier (BBB), protects the brain from toxic endogenous and exogenous substances, regulates blood flow, and provides growth factors needed to support proper neuronal development. The barrier mechanisms of the human, rat, and sheep brain are present from very early in gestation [18–23]. The fetal and neonatal BBB are effective from early in development and possesses many characteristics of the barrier observed in the fully developed adult brain [18–23].

Inflammation has long been known to promote BBB disruption and cerebrovascular dysfunction [24–27]. Inflammatory signals and neurovascular dysfunction can both result in neuronal damage [25, 28, 29]. Nonetheless, the effects of inflammation on the response of the NVU in the immature brain remain to be determined. Lipopolysaccharide (LPS) is a gram-negative bacterial endotoxin that is widely used as a robust inflammatory catalyst to stimulate neuroinflammatory responses in a variety of animal models including fetal sheep [10–15]. Single intravenous doses of LPS administered to fetal sheep resulted in extravasation of albumin into the brain parenchyma suggesting BBB disruption [30]. Similarly, injection of LPS into the uterine artery of pregnant ewes resulted in extravasation of plasma albumin into the cerebellar parenchyma of the fetus suggesting that maternal exposure to LPS also can compromise the fetal BBB [31]. Furthermore, exposure to LPS during development alters capillary density and interferes with angiogenesis in microvasculature of the retina, which is a vasculature bed that is structurally similar to the BBB [32, 33]. Taken together, these findings suggest that the neurovasculature in premature subjects could be vulnerable to inflammatory insults.

The overall goal of the current study was to test the hypothesis that prolonged exposure to inflammation adversely affects the neurovasculature in the brain of preterm fetal sheep. Therefore, we examined the effects of a prolonged exposure to a regimen of LPS on the quantity of blood vessels in the brain and changes in the NVU in fetal sheep in order to simulate exposure to in utero inflammation.

## Methods

### Animal preparation, study groups, and experimental design

All procedures were approved by the Animal Ethics Committee of the University of Auckland and carried out in accordance with the New Zealand Animal Welfare Act, and the Code of Ethical Conduct for animals in research established by the Ministry of Primary Industries, Government of New Zealand. The tissue samples for this study were obtained from animals, in which physiological changes and white matter injury have been previously reported [14, 15, 34, 35].

#### Fetal surgery

Singleton Romney/Suffolk fetal sheep were surgically instrumented at 98–100 days of gestation (term = 147 days) as previously reported for the purpose of the original studies [14, 15, 34, 35]. The ewes were given long-acting oxytetracycline (20 mg/kg, Phoenix Pharm, Auckland, New Zealand) intramuscularly 30 min before the onset of surgery. Anesthesia was induced by an intravenous injection of propofol (5 mg/kg; AstraZeneca Limited, Auckland, New Zealand) and maintained using 2–3% isoflurane in oxygen (Bomac Animal Health, NSW, Australia). During surgery, ewes received an intravenous infusion of normal (0.9%, w/v) saline (250 mL/h) to maintain an appropriate fluid balance. The depth of anesthesia, maternal heart rate, and respiration were continuously monitored by trained anesthetic staff.

A midline incision was made to expose the uterus, and the fetus was partially exteriorized for instrumentation. Polyvinyl catheters were placed in the left femoral artery and amniotic sac for the original studies. Catheters were also placed in the right brachial artery and left femoral vein for the intravenous infusions of LPS and normal (0.9%, w/v) saline.

All fetal catheters were exteriorized through the maternal flank, and a maternal saphenous vein was catheterized for post-operative care and euthanasia. Antibiotics were administered into the amniotic sac (gentamicin, 80 mg, Pharmacia and Upjohn, Rydalmere, NSW, Australia) before the uterus was closed. Ewes were given 5 mL of Streptocin (procaine penicillin, 250,000 IU/mL, and dihydrostreptomycin, 250 mg/mL, Stockguard Labs, Hamilton, New Zealand) intramuscularly 30 min before surgery for prophylaxis. The maternal midline skin incision was infiltrated with local analgesic (10 mL 0.5% bupivacaine plus adrenaline, AstraZeneca Ltd., Auckland, New Zealand).

#### Post-operative care

After surgery, ewes were housed together in separate metabolic cages with ad libitum access to food and water. Rooms were temperature and humidity controlled (16 ± 1 °C, humidity 50 ± 10%) with a 12-h light/dark cycle (light 06:00 to 18:00 h). Ewes were given daily intravenous antibiotics (600 mg Crystapen, Biochemie, Vienna, Austria and Gentamicin, 80 mg, for 4 days after surgery. Fetal catheters were maintained patent with continuous infusions of heparinized normal (0.9%, w/v; 20 U/mL at 0.2 mL/h).

### Experimental protocol

**Fig. 1:**
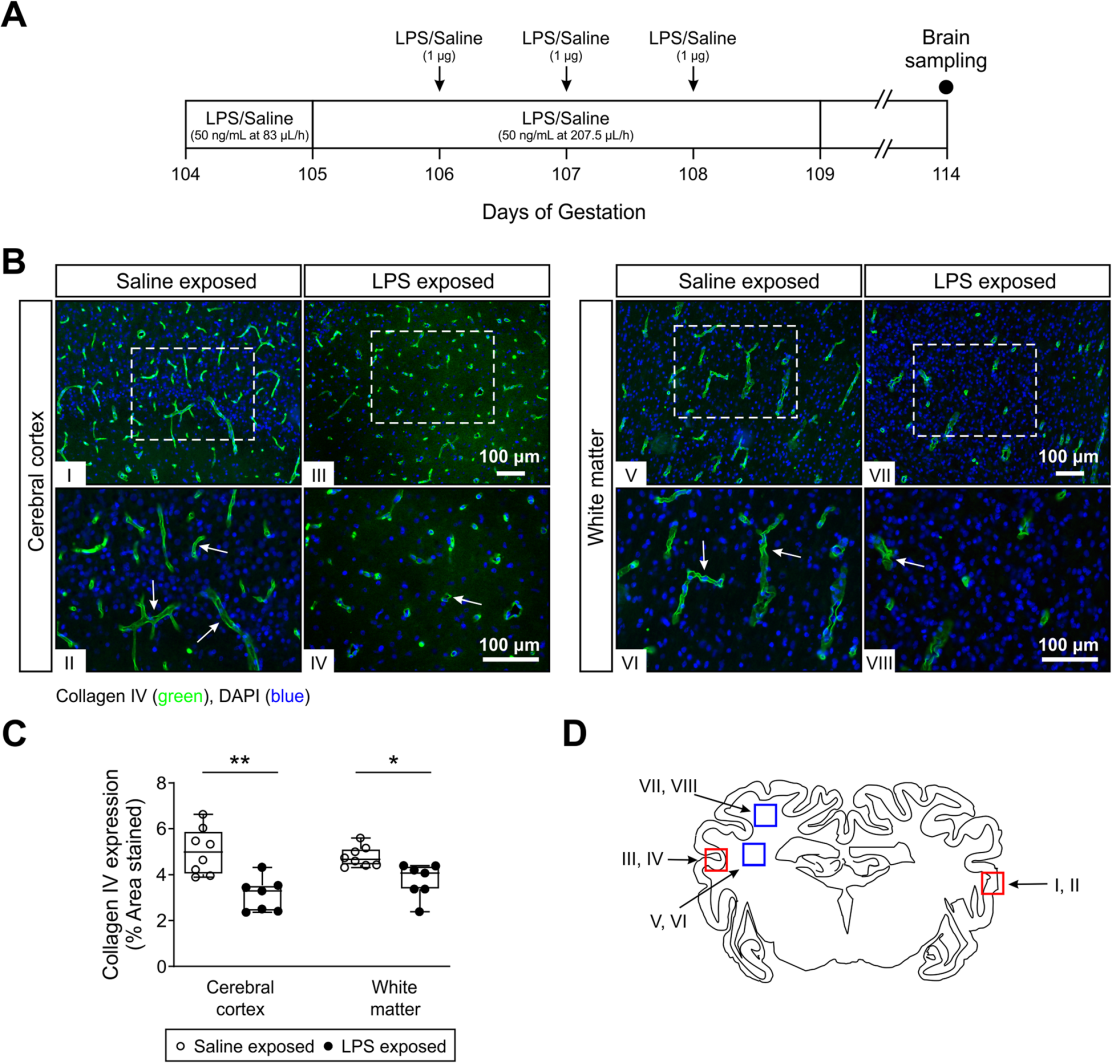
Schematic illustration of the study design and quantification of the microvasculature in randomly selected areas of preterm fetal sheep cerebral cortex and white matter from saline control and LPS-treated subjects. **a** After recovery from surgery at 103–104 days of gestation, the preterm fetal sheep were given either normal saline infusions/boluses (saline controls, *n* = 8) or LPS infusion/boluses (*n* = 7). LPS was infused at 100 ng/kg (50 ng/mL at 83 μL/h) for the first 24 h followed by 250 ng/kg/24 h (50 ng/mL at 207.5 μL/h) for the next 96 h. Boluses were administered as 1 μg LPS at 48, 72, and 96 h from the start of infusion. Normal saline controls received equivalent volumes of saline for both infusions and boluses. Ten days after the start of the infusions at 113–114 days of gestation, the fetal brains were collected for further immunohistochemical analysis. **b** Representative images of collagen type IV staining (green) in the cerebral cortex and white matter of saline control and LPS-exposed animals. × 10 (top row) and × 20 (bottom row) magnifications are shown, scale bar = 100 μm. White arrows indicate collagen IV-positive microvessels. DAPI (blue) is utilized as a counterstain. **c** Graphs representing the microvessel density expressed as the percent area of the fields (total area) segmented on anti-collagen type IV-stained sections in the cerebral cortex and white matter of the saline and LPS-exposed fetal sheep. An average of 15–20 fields on two slides per animal and *n* = 8 in the saline-exposed group and *n* = 7 in the LPS-exposed group were analyzed. Statistical analysis by Kruskal-Wallis test followed by Dunn’s post hoc test, *P < 0.05; **P < 0.01. **d** A schematically represented coronal cross-section of the sheep brain. The Roman numeral numbered boxes indicate the areas from which the images were taken in **b** and quantified in **c**

The experiments commenced 5 days after recovery from surgery at 103–104 days of gestation. This time in gestation is approximately comparable neuroanatomically to that of the preterm human brain between 28 and 32 weeks of gestation [36]. It represents 70% of the ovine gestation because full-term gestation in this breed of fetal sheep is 147 days. Hereafter, we refer to this time in gestation as 70% of gestation. Figure 1a shows a schema of the study design. Fetuses were randomly assigned to either (1) chronic normal saline infusions and saline boluses (saline controls, *n* = 8, 3 females and 5 males) and (2) chronic LPS (055:B5, Sigma Aldrich, St. Louis, MO, USA) infusion and LPS boluses (*n* = 7, 2 females and 5 males). LPS was dissolved in normal saline and infused at 100 ng/kg (50 ng/mL at 83 μL/h) for the first 24 h followed by 250 ng/kg/24 h (50 ng/mL at 207.5 μL/h) for the next 96 h. Boluses were administered as 1 μg LPS dissolved in 1 mL of normal saline at 48, 72, and 96 h from the start of infusion. Normal saline controls received equivalent volumes of saline for both the infusions and boluses. This LPS regimen was intended to produce a prolonged exposure to the inflammatory stimulus [14, 15, 34, 35] to simulate conditions similar to those in human pregnancies with prolonged rupture of membranes and chorioamnionitis and also to limit LPS-related fetal demise [37, 38]. Ten days after the start of the infusions at 113–114 days of gestation, the ewe was euthanized with an overdose of pentobarbital sodium (Pentobarb 300, Chemstock international, Christchurch, New Zealand). The fetal brain was perfused with heparinized normal saline (20 IU heparin/500 ml saline) followed by 1 L of 10% neutral buffered formalin. The brains were fixed for an additional 7 days in 10% neutral buffered formalin and divided into 4 to 5 mm thick coronal sections using a sheep brain slicer matrix and embedded in paraffin.

### Immunochemical staining and microscopy

Immunohistochemical analysis was performed on 10 μm deparaffinized and rehydrated coronal brain sections. Sections were prepared for glial fibrillary acidic protein (GFAP) and desmin plus collagen type IV double immunofluorescence as follows. The brain sections were immersed in EDTA buffer (1 mM EDTA, Sigma Aldrich, 0.05% Tween 20, Sigma Aldrich; pH = 8) and heated for 15 min in a pressure cooker in a microwave. The slides were rinsed with Tris-buffered saline (TBS, 10 mM, Sigma Aldrich) containing 0.025% triton-X100 (TBS-T, Sigma Aldrich) twice for 5 min each after the heat-induced epitope retrieval. The brain sections were then blocked in SuperBlock T20 (TBS) blocking buffer for 2 h at room temperature. The two primary antibodies were incubated overnight on two sequential days. First, the anti-GFAP (1:1000 dilution, Invitrogen, Carlsbad, CA, USA) or the anti-desmin (1:100 dilution, Invitrogen) were incubated overnight at 4 °C. On the second day, the slides were rinsed twice in TBS for 5 min and then the sections were incubated with collagen type IV antibody (1:100 dilution, Origen, Rockville, MD, USA) at 4 °C overnight to visualize the brain microvessels. On the next day, the slides were rinsed with TBS-T twice for 5 min, and then TBS and incubated with the appropriate Alexa Fluor 594- and 488-conjugated secondary antibodies (1:1000 dilution, Invitrogen) for 1 h in the dark at room temperature. The slides were then rinsed with TBS-T twice for 5 min then TBS and sections were subsequently cover slipped with DAPI containing mounting medium (Vector Laboratories, Burlingame, CA, USA). Staining for microglia was performed on sections that were immersed in citrate buffer (0.1 M citrate buffer, pH 6) and heated for 15 min in a pressure cooker to achieve antigen retrieval. The sections were then rinsed in TBS-T and blocked for 2 h in SuperBlock T20 (TBS) blocking buffer (Thermo Scientific, Wilmington, DE, USA). They were then incubated with anti-ionized calcium-binding adaptor molecule 1 (Iba-1) antibody (1:1000 dilution, Invitrogen) at 4 °C overnight. Thereafter, the slides were rinsed and incubated with Alexa Fluor 594-conjugated secondary antibodies (1:1000 dilution, Invitrogen) and then mounted as described above. Immunostaining without primary antibodies were used to rule out non-specific binding. All images were digitized and visualized using a Zeiss Axio Imager M2 Imaging System microscope (Carl Zeiss, Inc., Jena, Germany). The image acquisitions and analyses were performed on coded slides by two examiners (C.D. and F.A.), who were masked to the groups. Each investigator undertook the analysis separately. The final data represent the average values from both investigators. Simple adjustments were made to each entire image for the purpose of image presentation including adjustments in the contrast and intensity histogram strictly according to published guidelines [39].

### Quantification of single-labeled immunofluorescence

One brain section per animal was randomly selected for imaging and analysis. Fifteen to 20 fields per section were randomly selected for imaging and analysis based upon the collagen type IV staining of the blood vessels for the single-labeled immunohistochemical quantification of collagen type IV and GFAP in the cerebral cortical and white matter regions. The fields were systematically sampled in the cerebral cortical and white matter regions of the preterm fetal sheep brain such that an average area of 50 μm^2^ and 44 μm^2^ were sampled for cerebral cortex and white matter, respectively. Low and high power images were taken at × 10 and × 20, respectively. Images were segmented using an interactive thresholding modality, and the resulting binary images were processed with the measurement function of ImageJ software (NIH, Bethesda, MD). The immunoreactive positive areas were measured and quantified, and the final analysis expressed as an area fraction of the total area examined, which is the percent of the total field area that had been highlighted in green for collagen type IV or red for GFAP. Only blood vessels larger than 50 μm were used for the purpose of analysis.

### Quantification of pericyte and astrocytic blood vessel coverage

Multichannel channel images were acquired in order to quantify the blood vessel coverage by astrocytes and pericytes. Randomly selected vessels were imaged at × 40 magnification in the cerebral cortex or white matter based upon the collagen type IV staining. The channels were then split into the channels for the GFAP (red) or desmin (red) channels and the collagen IV (green) channel. The collagen IV source images were then thresholded to define the outer margin of the blood vessel under consideration and to measure the total blood vessel area. The red channel (GFAP or desmin) was then subtracted from the collagen IV (green) channel using the ImageJ image calculator tool. The same threshold used for the collagen IV was used for the resulting image and the area overlapping the red for the astrocytes or pericytes over the green blood vessel area was determined. The vascular coverage for the astrocytes and pericytes was expressed as a percentage of the total blood vessel surface area.

Quantitative image analysis was performed by two independent observers (C.D. and F.A.), who were not aware of the of the group designations. The two observers demonstrated excellent agreement for the astrocytic and pericytic microvascular coverage, *r* = 0.89, *n* = 15, *P* < 0.0001 and *r* = 0.95, *n* = 15, *P* < 0.0001, respectively. The final results represented the average of the mean values by the two independent observers. One brain section per animal and 15–20 fields randomly selected fields per section were analyzed in the cerebral cortex and white matter for all experiments in each brain of the fetuses from the control (*n* = 8) and LPS (*n* = 7) treated fetal sheep for each protein of interest.

### Statistical analysis

Results are presented in the text as mean and standard deviation of the mean. Box-and-whisker plots depict the median, 10th, 25th, 75th, and 90th percentiles as vertical boxes with error bars. Statistical analyses were performed using Graphpad Prism® (GraphPad Software, Inc, San Diego CA). Kruskal-Wallis test followed by Dunn’s post hoc test was used to compare the differences between the two groups. Linear regression analyses were used to determine correlations between GFAP and the vessel density; astrocytic coverage and the vessel density and pericyte coverage and the vessel density. *F* test was used to calculate the *P* values for the linear regression analyses. Differences were considered statistically significant if *P* < 0.05.

## Results

### Exposure to LPS reduces microvascular vessel density in the preterm fetal brain

Collagen type IV is a major molecular component of the vascular basal lamina, which we have previously demonstrated to be an excellent marker of the microvasculature in the fetal sheep brain [40]. Therefore, collagen type IV staining was used to visualize the microvascular network in the preterm fetal sheep cerebral cortex and white matter 10 days after the start of exposure to saline or LPS (Fig. 1). The number of collagen type IV green stained microvessels were greater in the cerebral cortex and white matter of the saline (Fig. 1b) than the LPS exposed fetal sheep. Analytic quantification of collagen type IV staining at low magnification (Fig. 1c) revealed a reduction in the quantity of the collagen type IV-immunostained microvessels in the cerebral cortex and white matter of the LPS compared with the saline exposed control fetal sheep. The percentage area stained with collagen IV in the cerebral cortex was 3.13 ± 0.73% in the LPS compared with 5.02 ± 1.00% in the saline exposed group. Similarly, the microvessel density was also lower in the white matter of the LPS, 3.7 ± 0.7%, compared with the saline, 4.8 ± 0.4%, exposed fetal sheep. The cross area of the sheep brain is schematically represented in Fig. 1d. The boxes designated by Roman numerals indicate the areas from which the images were obtained in Fig. 1b and quantified for the graphical analysis in Fig. 1c.

### Exposure to LPS reduces pericyte and vascular smooth muscle microvascular blood vessel coverage in the preterm fetal sheep brain

**Fig. 2:**
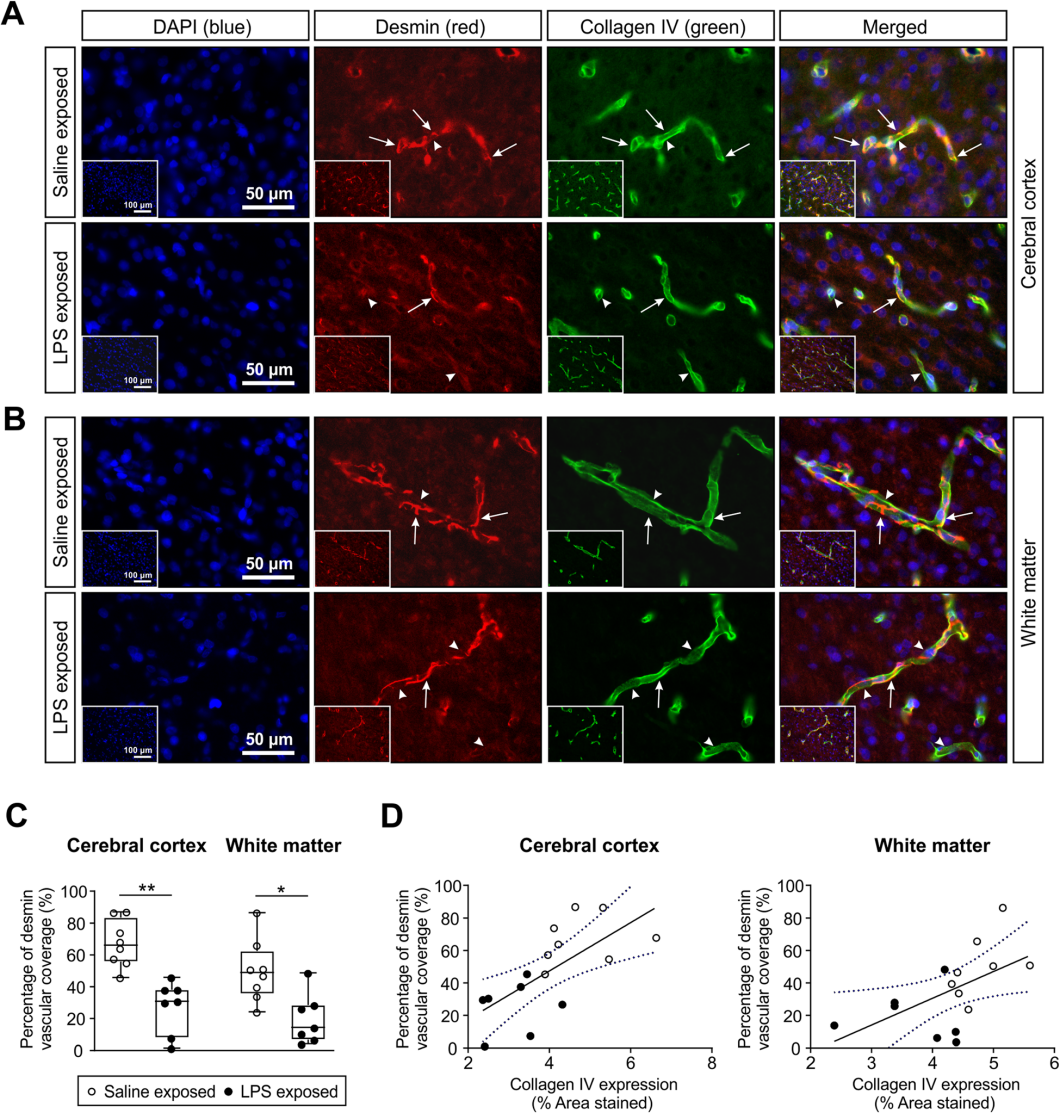
Pericyte coverage surrounding the brain microvessels. Representative images of desmin/collagen type IV double immunofluorescence labeled pericytes (desmin, red) and vessel basal lamina (collagen type IV, green) in the cerebral cortex (**a**) and white matter (**b**) of saline-exposed (top row) and LPS-exposed (bottom row) animals ( ×40 magnification scale bar = 50 μm, nuclear counterstaining DAPI (blue)). The inset (× 10 magnification, scale bar = 100 μm) on each image identifies the area of the brain from which the image was taken. White arrows indicate pericyte vascular coverage, whereas white arrowheads indicate the microvessels that are not covered by pericytes. **c** Graphs represent the quantification pericyte coverage in the cerebral cortex and white matter of the saline- and LPS-exposed fetal sheep. Pericyte coverage expressed as the percent of positive desmin area overlapping with the vessel wall. An average of 15–20 fields per region and per animal and *n* = 8 in the saline-exposed group and *n* = 7 in the LPS-exposed group were analyzed. Statistical analysis by Kruskal-Wallis test followed by Dunn’s post hoc test, *P < 0.05; **P < 0.01. **d** Correlations between pericyte microvessel coverage and vessel density in the cerebral cortex and the white matter of the fetal ovine brain. Desmin vascular coverage plotted on the *y*-axis against the collagen type IV expression as the vascular density in the cerebral cortex (*r* = 0.68, *n* = 15, *P* = 0.0052) and white matter (*r* = 0.56, *n* = 15, *P* = 0.031). Solid line is the regression line. Dashed lines are the 95% confidence intervals

Desmin is a muscle-specific class III intermediate filament found in pericytes and vascular smooth muscle cells that ensheath the microvasculature [41]. Pericyte and vascular smooth muscle cell coverage of the microvessels were examined by double immunostaining for desmin and collagen type IV in the cerebral cortical and white matter regions of the fetal sheep brain (Fig. 2a, b). The pericyte and vascular smooth muscle desmin coverage of the microvessels was significantly lower in the cerebral cortex and white matter of LPS compared with saline-exposed fetal sheep (Fig. 2a, b).

In the saline-exposed control group, we observed extensive desmin staining of the vascular walls corresponding to 66.9 ± 14.8% of the vascular coverage in the cerebral cortex and 49.5 ± 19.4% in the white matter (Fig. 2c). In the LPS exposed group, we observed significant reductions in the pericyte coverage corresponding to 40% in both cerebral cortex and white matter regions (Fig. 2c). In addition, we found a significant correlation between the desmin pericyte coverage and microvessel density in both the cerebral cortex (*r* = 0.68, *n* = 15, *P* = 0.005, Fig. 2d) and white matter (*r* = 0.56, *n* = 15, *P* = 0.031, Fig. 2d) supporting the concept of interrelated deficiencies in the constituents of the neurovascular unit.

### Exposure to LPS results in astrocyte activation and reduces astrocytic microvessel coverage in the white matter

**Fig. 3:**
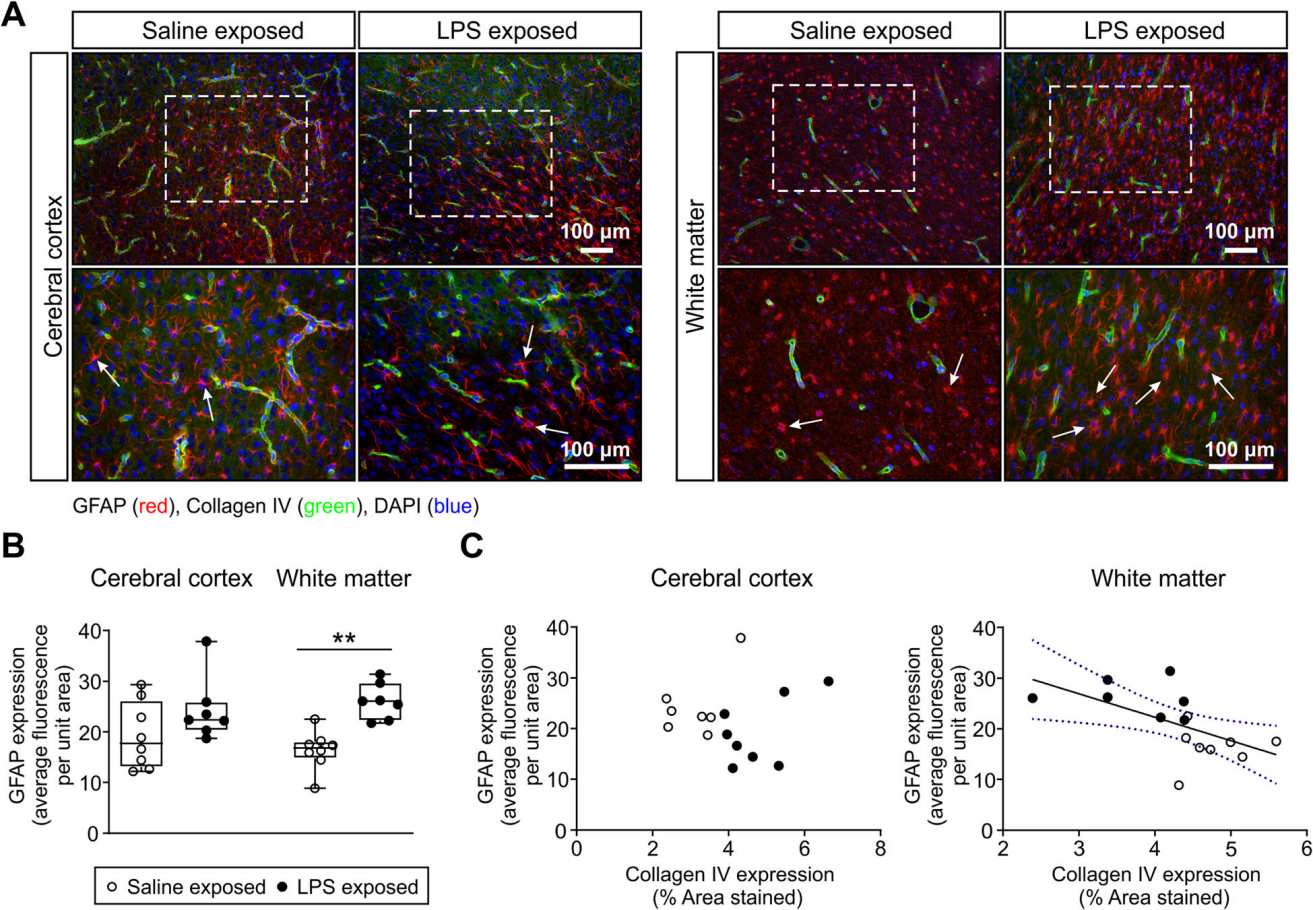
Astrocyte activation. **a** GFAP/collagen IV double immunofluorescence labeled astrocytes (GFAP, red) and microvessel basal lamina (collagen IV, green) in the cerebral cortex and white matter of saline- and LPS-exposed animals [× 10 magnification (top row) and × 20 magnification (bottom row), scale bar = 100 μm; nuclear counterstaining DAPI (blue)]. White arrows indicate GFAP-positive cells (**a**). **b** represent the quantification of GFAP immunoreactivity in the cerebral cortex and white matter of the saline- and LPS-exposed fetal sheep. GFAP expression as the percent of the area of the fields (total area) segmented on anti-GFAP-stained sections in the saline- and LPS-exposed groups. An average of 15–20 fields per region and per animal and *n* = 8 in the saline-exposed group and *n* = 7 in the LPS-exposed group were analyzed. Statistical analysis by Kruskal-Wallis test followed by Dunn’s post hoc test, **P < 0.01. **c** Correlations between perivascular astrocytes profiles and microvessel density in the cerebral cortex and white matter of the fetal ovine brain. GFAP expression profiles plotted on the *y*-axis against the collagen type IV expression as the vascular density in cerebral cortex (*r* = 0.073, *n* = 15, *P* = 0.80) and white matter (*r* = 0.59, *n* = 15, *P* = 0.02). Solid line is the regression line. Dashed lines are the 95% confidence intervals

**Fig. 4:**
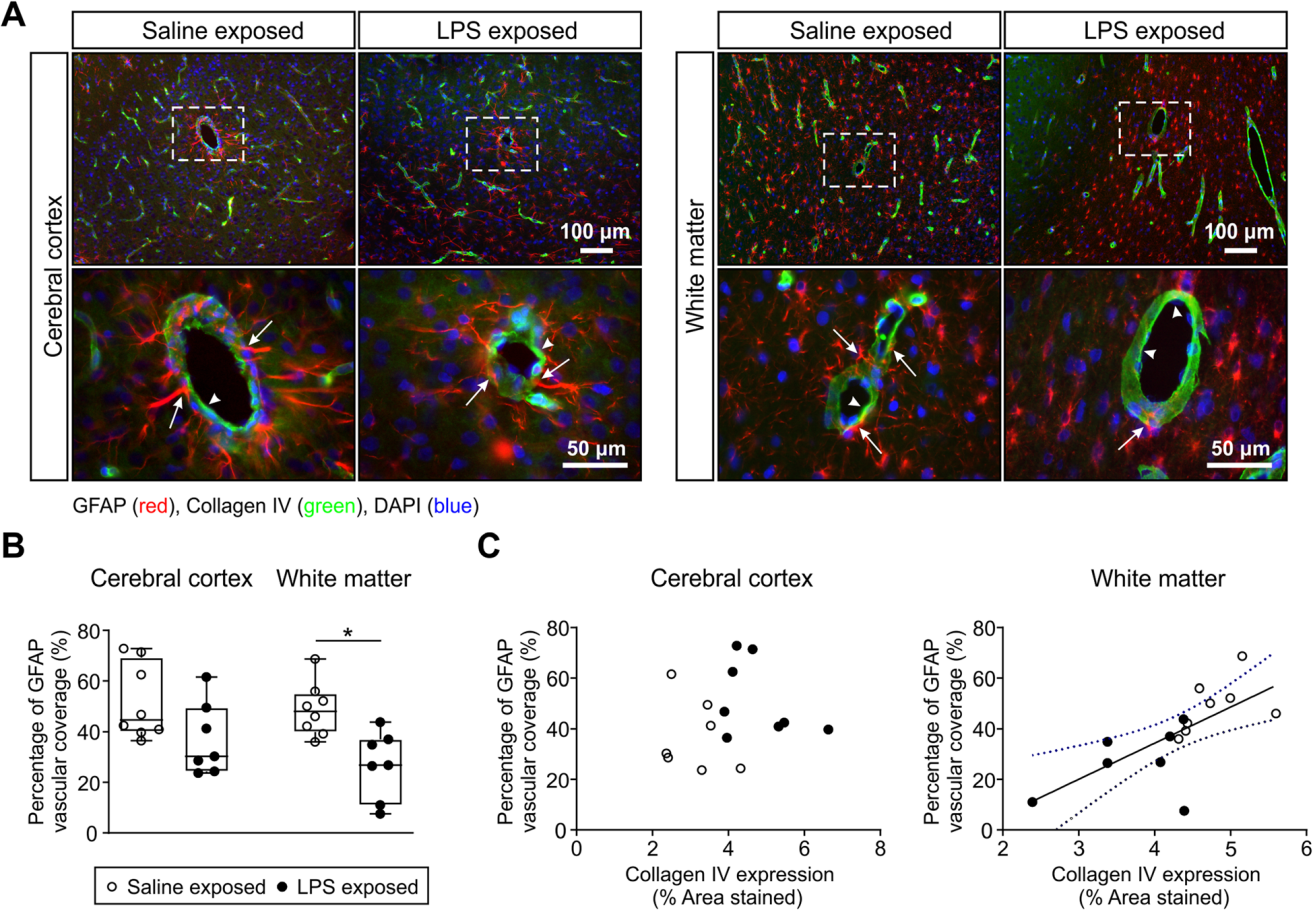
Astrocyte coverage surrounding the brain microvessels. **a** GFAP/collagen type IV double immunofluorescence, labeled astrocytes (GFAP, red) and microvessel basal lamina (collagen type IV, green) in the cerebral cortex and white matter of saline- and LPS-exposed animals [× 10 magnification (top row, scale bar = 100 μm) and × 40 magnification (bottom row, scale bar = 50 μm); nuclear counterstaining DAPI (blue)]. White arrows indicate astrocyte vascular coverage, whereas white arrowheads indicate the microvessels that are not covered by astrocytes. **b** represents the quantification of astrocytic end-foot coverage in cerebral cortex and white matter of the saline- and LPS-treated groups. Astrocytic coverage expressed as the percent of positive GFAP area overlapping the vessel wall. An average of 15–20 fields per region and per animal and *n* = 8 in the saline-treated group and *n* = 7 in the LPS-treated group were analyzed. Statistical analysis by Kruskal-Wallis test follow by Dunn’s post hoc test, *P < 0.05. **c** Correlations between perivascular astrocytes profiles and vessel density in the cerebral cortex and white matter of the fetal ovine brain. GFAP vascular coverage plotted on the *y*-axis against the collagen type IV expression as the vascular density in the cerebral cortex (*r* = 0.13, *n* = 15, *P* = 0.64) and white matter (*r* = 0.70, *n* = 15, *p* = 0.004). Solid line is the regression line. Dashed lines are the 95% confidence intervals

Double immunolabeling was performed with anti-GFAP as an astrocytic marker and anti-collagen type IV as a component of the basal lamina in both groups. The anti**-**GFAP antibody stained the astrocyte cell bodies, processes, and end-feet equally (Figs. 3a and 4a). Astrocytes exhibited a typical fine morphology with GFAP reactive processes that formed the end-feet surrounding the blood vessels in the saline-exposed fetal sheep brains (Figs. 3a and 4a). Astrocytes were stained in both the cerebral cortex and white matter. LPS exposure was associated with reactive type astrocytic morphology characterized by hypertrophy and swelling of the astrocyte body and processes (Figs. 3a and 4a). Exposure to the LPS regimen resulted in the activation of the astrocytes in the white matter (Figs. 3a and 4a). The morphological changes were not associated with significant increases in GFAP immunoreactivity in the cerebral cortex. The average cerebral cortical GFAP fluorescence per unit area was 19.3 ± 6.6 in the saline compared with 24.4 ± 6.4 in the LPS-exposed group (Fig. 3b). GFAP immunoreactivity in the white matter was significantly greater in the LPS compared with the saline-exposed group suggesting the presence of reactive astrogliosis. The GFAP average fluorescence per unit area in the white matter was 16.4 ± 3.9 in the saline versus 26.1 ± 3.5 in the LPS-exposed group (Fig. 3b). The GFAP expression per unit area showed an inverse correlation with collagen type IV expression in the white matter (*r* = 0.58, *n* = 15, *p* = 0.022, Fig. 3c), but not in the cerebral cortex (Fig. 3c).

The overlap of GFAP and collagen type IV was quantified to evaluate astrocyte end-feet coverage surrounding the brain microvessels (Fig. 4). As expected in the saline-exposed group, astrocyte end-feet were present surrounding the brain microvessels within the cerebral cortex and white matter, indicating close contact between the glia limitans and the microvasculature (Fig. 4a). The average astrocytic coverage in the saline group was 51.7 ± 14.9% of the vascular area in the cerebral cortex and 48.8 ± 10.5% in the white matter (Fig. 4b). The development of hypertrophic end-foot processes was associated with significant decreases in the overlap of GFAP and collagen type IV in the white matter of the LPS-exposed group, 26.8 ± 13.4% of the vascular area, but not in the cerebral cortex, 37.1 ± 14.3% of the vascular area in the cortex. This suggests that exposure to LPS diminished the close contacts between the vasculature and astrocytes surrounding the microvessels in the white matter (Fig. 4b). In addition, we demonstrated a direct correlation between the astrocyte vascular coverage and the vascular density (*r* = 0.69, *n* = 15, *P* = 0.0041, Fig. 4c) in the white matter. However, a significant correlation was not observed in the cerebral cortex for the same analysis (Fig. 4c).

### Exposure to LPS results in microglial activation

The LPS-related inflammatory signals predispose to increases in BBB permeability and facilitate the entry of macrophages and cytokines from the blood into the brain to further accentuate neuroinflammation [42–45]. We used Iba-1 as a marker in this study because it recognizes the ramified cellular shape of the microglia. The resting-type microglia were characterized in the saline-exposed control fetal sheep by a ramified phenotype evenly distributed in the perivascular space of the cerebral cortex and white matter (Supplemental Figure 1A and B upper panel). We confirmed the presence of activated amoeboid microglia in perivascular spaces in the white matter of LPS exposed animals (Supplemental Figure 1B lower panel). By contrast, there were no morphological changes of microglia in the cerebral cortex of LPS exposed fetal sheep (Supplemental Figure 1A lower panel) consistent with the previous report in the same animal model [14].

In the current study, we also attempted to co-label the microvasculature along with microglia in order to quantify the microglia that were associated with the microvasculature. However, even after attempting to stain the microvessels with collagen type IV along with the microglia using four different antibodies (anti-Iba1 antibody, chicken polyclonal 139590, Abcam, Cambridge, MA, USA; anti-Iba1 antibody, mouse monoclonal 15690, Abcam, anti-Iba1 antibody, mouse monoclonal, 016-26721 FUJIFILM Wako, Richmond, VA, USA; and anti-Iba1 antibody, goat polyclonal, ab5076, Abcam) and several different heat antigen retrieval buffers, we were not able to obtain reliable results simultaneously with the two stains in the fetal sheep brain tissue. Therefore, we were unable to quantify microglia associated with microvessels.

## Discussion

Inflammation has been shown to be associated with impaired brain development and to predispose to neurodevelopmental abnormalities and disabilities in late childhood [46, 47]. The purpose of the current study was to test the hypothesis that inflammation adversely affects multiple components of the NVU in the cerebral cortex and white matter of the preterm ovine fetus after exposure to a prolonged inflammatory stimulus in utero. We demonstrate first that exposure to prolonged inflammation was associated with significant decreases in the microvascular density in the cerebral cortex and white matter of the ovine fetal brain 10 days after the onset of exposure to LPS. Secondly, pericyte microvascular coverage was decreased in the cerebral cortex and white matter. Thirdly, we observed increased astrogliosis, associated with decreases in the astrocyte end-feet coverage surrounding the microvasculature in the white matter.

This study examined fetal sheep at 103–104 days of gestation, which represents approximately 70% of the ovine gestation. The neuroanatomical development of the brain of the ovine fetus at this time in gestation is approximately similar to the preterm human brain between 28–32 weeks [36, 48, 49]. The neurodevelopment of the immature ovine brain is also similar to that of the premature infant with respect to the completion of neurogenesis, onset of cerebral sulcation, and emergence of the cortical components of the auditory and somatosensory evoked potentials [36, 50–53]. Consequently, studies of the vasculature in the brain of the preterm fetus at this time in gestation are relevant to understanding the development of brain injury in preterm infants.

Treatment of neonatal rats with LPS has also been reported to result in white matter damage and acute transient disruption of the BBB [26]. In addition, LPS exposure has previously been suggested to increase BBB permeability in the ovine fetus [30, 31]. These studies are consistent with findings suggesting that LPS-induced inflammation has important effects on the cerebral vasculature during fetal development [26, 30, 31].

Inflammation of the neurovasculature is associated with activation of all cellular constituents in the NVU and with release of endogenous factors in brain cells. Therefore, we hypothesized that systemic inflammation could disrupt angiogenesis in the brain during development and alter the morphology of the NVU. Immunohistochemical analysis of the microvasculature after exposure of preterm fetal sheep to LPS in the present study demonstrated that the mean microvessel density was reduced in both the cerebral cortex and white matter. Vessel density was determined by measuring collagen type IV immunoreactivity. We have previously identified neovascularization within 72 h after the exposure of the fetal sheep brain to ischemia-reperfusion using the same methodology and marker [40]. Therefore, the immunohistochemical and morphometric analysis in this and our recent work suggests that collagen type IV is an excellent measure of microvessel density in the brain of the ovine fetus [40]. Collagen type IV is a major component of the basal lamina that provides structural stability to the vessel wall. Therefore, our findings suggest that prolonged exposure to LPS results in either a breakdown in the basal lamina and/or a reduction in vessel growth in the brain of the preterm fetus.

Pericytes are critical cellular components of the NVU that are central to the development and maintenance of the BBB because of their contributions to the regulation of the endothelial cellular junctions [22, 54–56]. Pericytes participate in angiogenesis and perform cellular immune functions in addition to their contribution to the structural integrity and stability of the neurovasculature [56]. Coverage of the microvasculature by pericytes in the premature human cerebral cortex and white matter was approximately 90% using neural/glial antigen 2 (NG2) and platelet-derived growth factor receptor beta (PGDFR-β) as pericyte markers along with CD34 as an endothelial marker [57] compared to approximately 67 and 50% in the cortex and white matter, respectively, in control preterm fetal sheep in the present study. The lower pericyte coverage in the control preterm fetal sheep brain compared with the human brain could be related to species differences and/or pericyte marker differences and the use of CD34 as an endothelial marker versus collagen type IV as a basal lamina marker [57].

Exposure of the preterm fetal sheep to LPS resulted in significantly reduced pericyte coverage of the microvasculature in both the white matter and cerebral cortex. We speculate that the LPS-related reductions in pericyte coverage could result in vascular fragility, adverse consequences in BBB function, vascular contractility, and vessel growth and, consequently, alterations in brain perfusion and impaired brain growth and maturation. If similar exposure to inflammation to the preterm human fetus reduces pericyte coverage, this could in part contribute to the association between inflammation and increased risk of intraventricular hemorrhage and adverse neurological outcomes in premature infants [58, 59].

Astrocytes are essential to normal brain and cerebrovascular function [60]. The end-feet of astrocytes surround the brain microvasculature and regulate a wide variety of functions at the BBB, and so astrocytes are key elements of the NVU [61]. Abnormalities in astrocyte end-feet can have dramatic consequences on the integrity of the BBB resulting in the development of brain disorders. Furthermore, pericytes in conjunction with astrocyte end-feet facilitate the expression of endothelial tight junction proteins, transporters, and enzymes within the microvasculature [17, 62, 63]. Astrocyte end-feet support ion transport, metabolite and energy substrate exchange from the blood to brain [61]. In addition, astrocytes secrete a variety of vasoactive molecules that modulate vascular tone [64–66]. Injury to gliovascular coupling could disrupt the integrity of the BBB and alter BBB transport function [67, 68]. In the present study, inflammation induced by LPS exposure resulted in astrogliosis with typical end-feet swelling associated with a reactive morphology in both the cerebral cortex and white matter, with a significant overall increase in GFAP immunoreactivity in the white matter, consistent with previous reports [14, 35].

The coverage of astrocytes surrounding the brain microvessels in the control saline-exposed preterm fetal sheep was approximately 52% in the cerebral cortex and 49% in white matter. These findings are consistent with evidence in premature human infants at a similar stage of brain development using the same astroglial marker [69] supporting the contention that the neurovascular development of the preterm ovine fetal brain is similar to that of the preterm human brain between 28–32 weeks [18, 48, 49]. LPS exposure was associated with a reactive morphology of astrocytes and a significant decrease in the astrocytic end-foot coverage in the white matter. The loss of astrocytic and pericyte microvascular coverage after exposure to LPS and the consequent inflammatory stress-related condition suggests the potential for impaired gliovascular coupling that could adversely affect the BBB [30].

We have previously demonstrated that the pro-inflammatory cytokine IL-6 is increased in the blood and tumor necrosis factor-α (TNF-α) in the brain parenchyma of fetal sheep after exposure to LPS [14, 35]. Elevated cytokines within the brain parenchyma can originate locally from stimulated intrinsic cells and from infiltrated cells that originate in the systemic circulation and/or by crossing the BBB [70]. Microglia are the chief intrinsic contributors to the neuroinflammatory response among the intrinsic cellular elements in the brain [71, 72]. They populate the CNS very early in development, are present in all brain regions accounting for approximately 15% of glial cells [73], and contribute to synaptic remodeling, neuronal migration, and axonal growth during brain development [71, 72, 74, 75]. Microglia are also involved in angiogenesis and participate in NVU dynamics to maintain stability of the microenvironment and homeostasis of the brain parenchyma [71, 76].

Activated microglia migrate to the site of inflammation or of injury under inflammatory conditions to remove cellular debris and produce large quantities of pro-inflammatory mediators. We have previously reported and quantified infiltration of activated microglia into the periventricular white matter in LPS-exposed fetal sheep [14]. In our earlier report, the brains of the LPS-exposed fetal sheep showed patchy infiltration of activated microglia using isolectin B4 as the stain [14]. The numbers of microglia were significantly greater in the perivascular white matter compared with the control fetal sheep [14]. In the current study, we confirmed this observation by using Iba-1 as the microglia marker. Unfortunately, we were not able to quantify the microglia activation associated with the microvasculature because of technical issues related to the simultaneous staining of both the microvasculature and microglia in the sheep brain.

The consequent inflammatory signals predispose to increases in BBB permeability and facilitate the entry of macrophages and cytokines from the blood into the brain to exacerbate neuronal damage. We used Iba-1 as a marker in this study because it recognizes the ramified cellular shape of the microglia. Resting type microglia were characterized in the saline control fetal sheep by a ramified phenotype evenly distributed in the perivascular space of the cerebral cortex and white matter (Supplemental Figure 1A and B upper panel). We confirmed the presence of activated amoeboid microglia in perivascular spaces in the white matter of LPS-exposed animals (Supplemental Figure 1B lower panel). By contrast, there were no morphological changes of microglia in the cerebral cortex of LPS-exposed fetal sheep (Supplemental Figure 1A lower panel).

The morphological alterations in the NVU in the cerebral cortex and white of preterm ovine brain after exposure to LPS in the present study likely contribute to inflammation-related brain injury. This postulate is supported by the close correlation between the responses of the various cellular components of the NVU and vessel density, further supporting the contribution of cellular communication in angiogenesis during development. Dysregulation in the components of the NVU could provide a therapeutic target to reduce the impact of inflammation-related disorders in the preterm brain. However, the precise timing of such modulations and the ability of the NVU to recover remain to be determined in future experiments. Furthermore, it will be important to determine whether the changes in the NVU are transient or result in prolonged and more persistent damage to the NVU.

Although previous studies have demonstrated that acute and chronic inflammation results in robust inflammatory responses (glial activation and cytokine release) and cell death (neuronal apoptosis), this is the first report to demonstrate significantly reduced microvessels in the cerebral cortex and white matter of fetuses exposed to prolonged inflammation [7, 29, 77, 78]. This decrease in the amount of cerebral microvessels in the brain of fetuses exposed to LPS could have an important impact on brain development because the vasculature provides the brain with oxygen, glucose, and nutrition. Therefore, examination of treatment strategies to prevent or attenuate the changes that we observed would be important. Although antibiotics and antenatal corticosteroids are routinely used in pregnancies with premature rupture of membranes and chorioamnionitis, we do not know if these strategies or potential future anti-inflammatory strategies could attenuate or eliminate the changes that we observed in the neurovasculature of the fetal sheep after exposure to LPS [38, 79–83]. Even though some anti-inflammatory strategies have been shown to attenuate inflammation and changes in the BBB in animal models [83–86], there are as yet no clinically established anti-inflammatory strategies to attenuate inflammatory changes in human pregnancies. Moreover, it remains to be determined whether treatment with antibiotics and/or anti-inflammatory strategies would reverse or attenuate the changes in the neurovasculature that we observed in the preterm fetal sheep.

## Conclusions

**Fig. 5:**
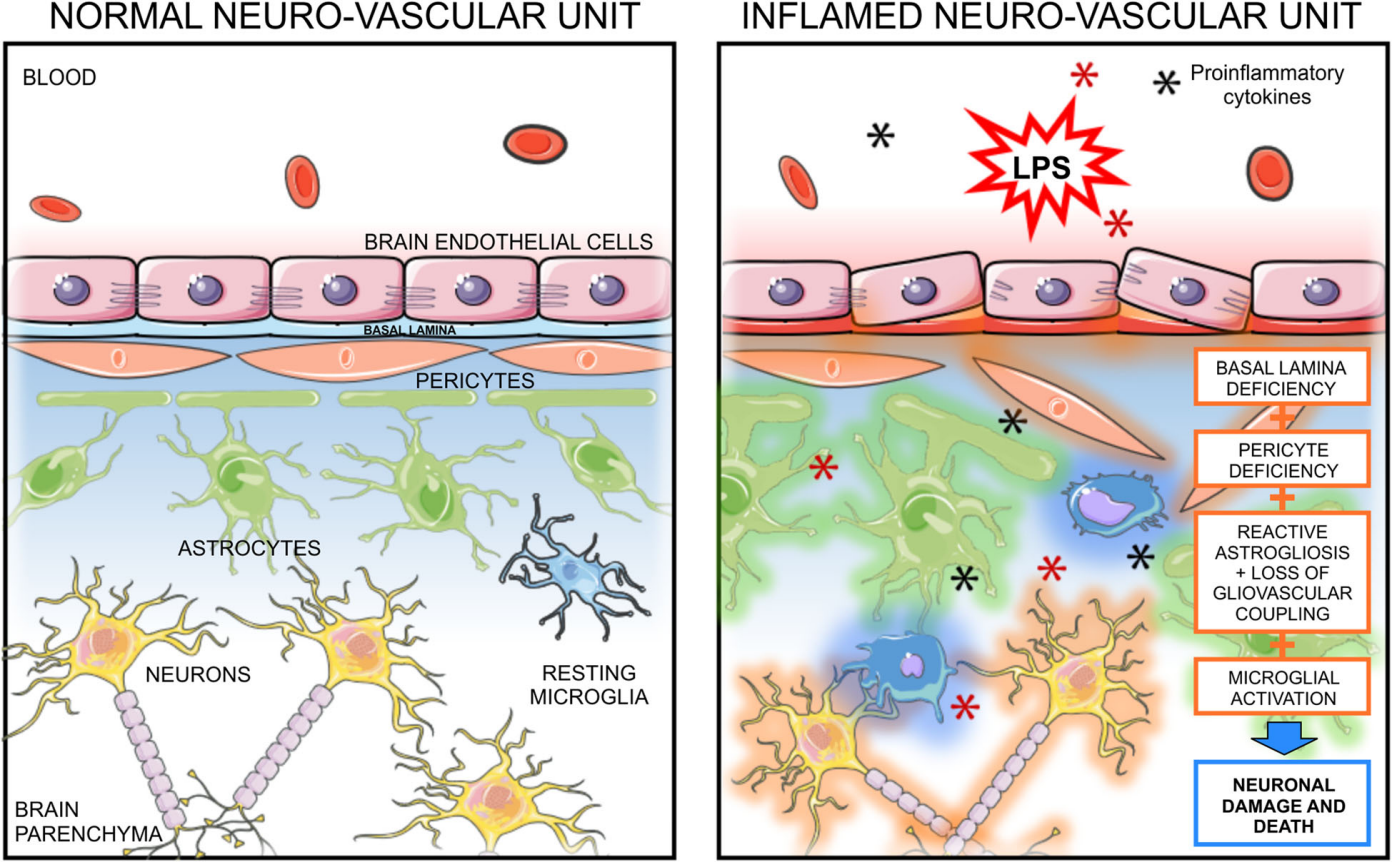
Schematic representation of the neurovascular unit in the saline control and LPS-exposed preterm sheep brain. The left-hand panel illustrates the components of the NVU in the normal brain. The cerebral vascular endothelium is intact and connected by tight junctions. Pericytes surround the vascular endothelium and astrocytes surround the pericytes and vascular endothelium. Resident neurons and microglia are present in the resting state. The right-hand panel schematically illustrates the effects of prolonged LPS exposure on the components of the NVU examined in the current study. The findings of the study are highlighted in the orange boxes. Systemic infusions of LPS resulted in reductions in the basal lamina and in the perivascular pericyte microvascular coverage, reactive astrogliosis, reductions in perivascular astrocytic microvascular coverage in white matter, and increases in activation of microglia. We speculate that these changes may predispose to impaired brain development after premature birth

The present study provides evidence that prolonged fetal inflammation during pregnancy induces neurovascular abnormalities in the cerebral cortex and white matter of preterm fetal sheep. The responses of the NVU to prolonged exposure to LPS in the preterm ovine fetus are schematically summarized in Fig. 5. In the normal fetus (left panel), the cerebral vascular endothelium is intact and connected by tight junctions. Pericytes surround the vascular endothelium and astrocytes surround the pericytes and vascular endothelium. Resident neurons and microglia are present in the resting state. After LPS exposure (right-hand panel), there is decreased cerebral cortical and white matter vascular density, and astrogliosis, reduced pericyte and astrocytic microvascular coverage, and microglial activation. We speculate that these alterations in the NVU could predispose to neuronal injury and impaired brain growth and maturation [7, 14, 15].

## Supplementary information

**Additional file 1: Figure S1.** Perivascular microglia activation. Representative images of Iba**-**1 staining (red) in the cerebral cortex (A) and white matter (B) of the saline (top row) and LPS (bottom low) exposed fetal sheep. 40 x magnification, Scale bar = 20 μm, nuclear counterstaining DAPI (blue). White arrows indicate Iba-1 positive microglia. VL= vessel lumen.

## Data Availability

All data generated or analyzed for this manuscript during this study is contained within the manuscript.

## Abbreviations

LPS: Gram-negativeendotoxin lipopolysaccharide
NVU: Neuro-vascular unit
BBB: Blood-brain barrier
GFAP: Glial fibrillary acidic protein
Iba-1: Ionized calcium-binding adaptor molecule 1
