# Supplementary figures and images for: Lipopolysaccharide-induced changes in the neurovascular unit in the preterm fetal sheep brain
Source: J Neuroinflammation. 2020 May 28;17:167. doi: 10.1186/s12974-020-01852-y (PMC7257152; doi:10.1186/s12974-020-01852-y)

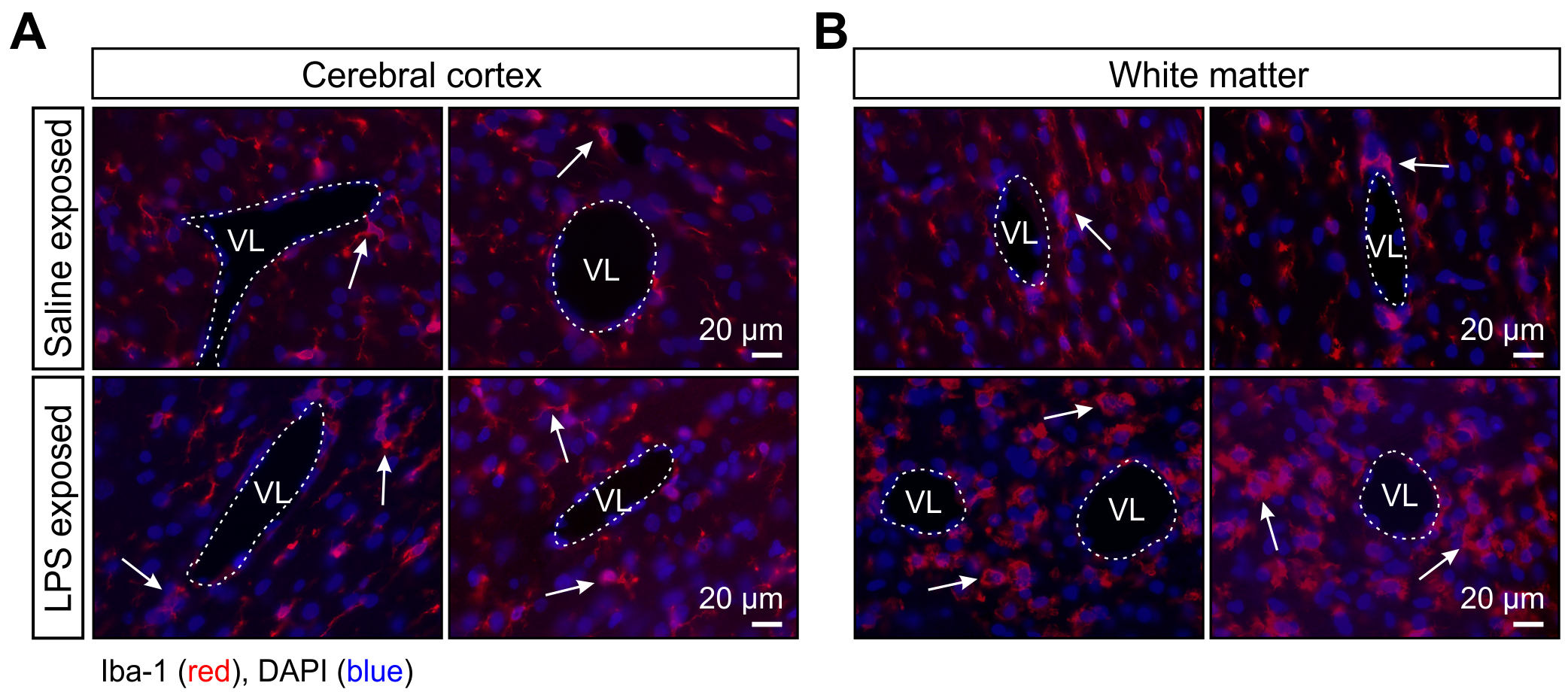

Supplement: Supplementary file 1 — Additional file 1: Figure S1. Perivascular microglia activation. Representative images of Iba-1 staining (red) in the cerebral cortex (A) and white matter (B) of the saline (top row) and LPS (bottom low) exposed fetal sheep. 40 x magnification, Scale bar = 20 μm, nuclear counterstaining DAPI (blue). White arrows indicate Iba-1 positive microglia. VL= vessel lumen. [file 12974_2020_1852_MOESM1_ESM.tif]
